# Our Encounters with Self-Harm

**DOI:** 10.1192/pb.bp.114.047126

**Published:** 2015-02

**Authors:** Elizabeth J. F. Hunt

**Figure:**
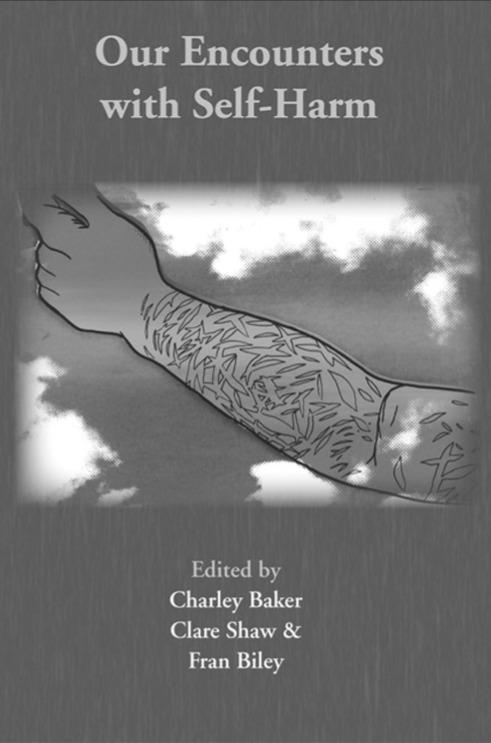


When I was working as a junior doctor in accident and emergency, one of my favourite jobs was suturing. A pleasant break from the constant decision-making, an opportunity to do something practical and almost artistic, and most of all a chance to just sit down and chat with the patient as I stitched. Despite this, one of my least favourite tasks was patching up those who had self-harmed. Something about the sight of the self-inflicted wounds upset me far more than the most horrendous accidental injuries; I tried to still be warm and not allow the distress and disgust I felt show, but I did not know what to say and we would often sit in silence as I worked. I had never been taught about non-suicidal self-harm, what it serves, how to approach it, anything. It was not until I began my psychiatric training that I began to understand it, and became retrospectively frustrated with how I had felt and responded to it earlier in my career. I now ensure I cover this subject in some depth with my medical students, to try to avoid them feeling about self-harm the way that I used to. Alternatively, I could just make them read this book.

*Our Encounters with Self-Harm* is made up of 37 pithy chapters by different authors. The majority are written by those who have, or still do, self-harm, and others are by family members and professionals (the last of which I found the least educational; an interesting reminder not to dismiss personal accounts in an era where quantitative research often feels the only thing that counts). Most take the form of a piece of prose about the writer’s personal experience, followed by a short bullet-point list of thoughts that they would like the reader to take away from it.

These pieces are brave, articulate, occasionally harrowing, and frequently illuminating. Since it is an anthology, unsurprisingly there is a certain amount of repetition within the book. This is no complaint; it serves to reinforce the most common themes such as: accept that this is my coping mechanism, find out what it means to me, look beyond the act of self-harm to treat the person behind it with kindness. Meanwhile, the divergences remind us of other key points such as not making assumptions and remembering that ‘everyone who self-harms is an individual, so everyone’s self-harm has individual meaning’.

Buy this book and force every medical and nursing student you encounter to read a chapter from it. I suspect it would significantly improve in the future the care that patients presenting to accident and emergency with self-harm receive. While you are at it, share it with your psychiatric colleagues; a reminder of empathy and a deepening of our understanding of our patients can never go amiss. As one of the contributors writes, ‘whatever you learn, get it out there, you never know who might benefit from your own experiences’.

